# Assessment of the *TGFB1* gene expression and methylation status of the promoter region in patients with colorectal cancer

**DOI:** 10.1038/s41598-022-15599-4

**Published:** 2022-07-07

**Authors:** Damian Wodziński, Agnieszka Wosiak, Jacek Pietrzak, Rafał Świechowski, Radzisław Kordek, Ewa Balcerczak

**Affiliations:** 1 Laboratory of Molecular Diagnostics and Pharmacogenomics, Department of Pharmaceutical Biochemistry and Molecular Diagnostics, Interfaculty Cathedral of Laboratory and Molecular Diagnostics, Medical University of Lodz, Lodz, Poland; 2 Department of Pathology, Cathedral of Oncology, Medical University of Lodz, Lodz, Poland

**Keywords:** Cancer, Molecular biology, Gastroenterology, Molecular medicine, Oncology

## Abstract

The aim of this study was to evaluate the expression of the *TGFB1* gene encoding the TGF-*β*1 cytokine in 64 patients, and then to compare it with clinico-pathological features. The study also investigated whether the regulation of the gene expression is caused by methylation of the promoter region between − 235 and + 22 nucleotide from the start of transcription. The dependence of the relative level of the *TGFB1* gene expression on the clinical advancement according to the TNM classifications was shown. Additionally, the individual grades of the T and M features of the TNM classification differed in the relative transcript levels of the *TGFB1* gene. Moreover, the higher relative expression level of the studied gene was associated with a lack of vascular invasion by cancer cells and presence of lymphocytes in the neoplastic tissue. The obtained results may indicate a possible impact of the gene on the process of carcinogenesis in colorectal cancer and reduction of its expression level may be one of the factors contributing to progression of the disease.

## Introduction

Despite the progress of civilization, colon cancer is still a serious health problem in the world, especially in highly developed countries. According to the statistics of the Global Cancer Observatory in 2020 there were almost two million new cases of colorectal cancer, which makes it the third most commonly diagnosed cancer in the world^1^. The huge scale of the problem prompts many researchers to explore the basis of carcinogenesis.

A small percentage of colon cancers are inherited like Lynch's syndrome or familial adenomatous polyposis (FAP). However, a vast majority of cases are sporadic colorectal cancers associated with older age, low physical activity, a high saturated-fatty acids diet and low fiber intake, as well as spontaneous changes at the molecular level^2,3^. Disturbances in the TGF-*β* signaling pathway are believed to be one of the factors associated with the development of colorectal cancer^4–7^.

The TGF-*β* signaling pathway plays a very important role in embryogenesis and cellular homeostasis^4,8^. It is involved in many biological processes such as control of cell growth, differentiation or migration, and even regulation of apoptosis^8,9^. The superfamily of the TGF-*β* signaling pathway consists of 40 different proteins. This group includes, among others, nodal growth differentiation factor (nodal), activin, inhibin, bone morphogenetic proteins (BMPs), growth and differentiation factors (GDFs), anti-Müllerian hormone (AMH) or TGF-*β* proteins^9–11^. These cytokines, with the participation of specific transmembrane receptors and cytoplasmic proteins, transmit a signal to the cell nucleus and regulate the expression of various genes^8,12^.

In the canonical TGF-*β* signaling pathway dependent on SMAD proteins, signal transduction begins with the binding of a dimeric ligand to a type II receptor (T*β*R-II) binding domain which initiates activation of its kinase domain. As a result of increasing the affinity to the type I receptor (T*β*R-I), a heterotetramer is created. It consists of two T*β*R-II and two T*β*R-I molecules^10,13^. This results in phosphorylation of the glycine and serine-rich GS domain of the type I receptor by T*β*R-II, which involves the cytoplasmic proteins of the SMAD family. These proteins enter the cell nucleus where they interact with DNA-binding proteins, transcriptional coactivators and corepressors to regulate the expression of target genes^4,8,10,14^.

Apart from the canonical pathway, the ligands of the TGF-*β* may also involve proteins of other signaling pathways in which the regulation of gene expression occurs without SMAD proteins. The pathways of mitogen-activated protein kinases (MAPKs) and phosphoinositide 3-kinases (PI3Ks) are recruited via TGF-*β* and bone morphogenetic protein receptors^8^. After binding to the TRAF6 (TNF receptor-associated factor 6), the TGF-*β* receptors activate the TAK1 protein (TGF-*β*-associated kinase 1) which activates the signaling cascades of the MKK4-JNK, the MKK3/6-p38 and the IKK-NFκB ones. Through them, the signal is also transmitted to other signaling molecules, such as PAR6, RhoA, Cdc42 or PAK2, which play a significant role in the process of the epithelial-mesenchymal transition^12,15,16^.

The TGF-*β* is one of the most important cytokines in the regulation of tissue development and homeostasis. It is released mainly by thrombocytes and, to a lesser degree, by activated macrophages, lymphocytes and neutrophils. Particularly high concentrations of the TGF-*β* are found at the site of an ongoing inflammatory reaction. In addition, the protein also participates in bone formation, angiogenesis, development of muscle and adipose tissue, inflammation and wound healing. Many reports indicate that dysfunctions of the protein are associated with fibrotic diseases, connective tissue disorders and cancers^11,17–19^.

The *TGFB1* gene encoding this cytokine is located on the long arm of chromosome 19 and is composed of seven exons and six introns with a total length of 52.3 kb^20,21^. The expression product of the gene is the pro-TGF-*β* protein with a molecular weight of 44.3 kDa and composed of 390 amino acids. The pro-TGF-*β* is a precursor of the LAP protein (latency associated peptide) and TGF-*β* (transforming growth factor *β*) belonging to the TGF-*β* superfamily ligands^22,23^. There are three isoforms of transforming growth factor *β*, i.e., TGF-*β*1, TGF-*β*2 and TGF-*β*3, which in the extracellular space exist in an inactive form, non-covalently associated with other proteins. As a result of the formation of the so-called small latency complex (SLC) and large latency complex (LLC), the ability to bind the cytokine to receptor proteins is lost. Due to the presence of complexes, there is often an excess of inactive TGF-*β* in the extracellular space which may be quickly used in the case of increased demand, e.g., during embryogenesis, wound healing, fibrosis or carcinogenesis^12^.

The involvement of the *TGFB1* gene in the progression of colorectal cancer has been extensively described. Under physiological conditions, the *TGFB1* gene acts as a tumor suppressor that activates apoptosis and lowers the expression of the gene encoding vascular endothelial growth factor^24^. In turn, it has been observed in many studies that overexpression of this gene is associated with the formation of neoplastic stem cells in the tumor stroma and a worse response to treatment^25^. Additionally, it promotes the epithelial-mesenchymal transition (EMT) and thus the formation of metastases^26,27^. It has also been documented that overexpression of the *TGFB1* reduces the immune response against cancer cells, which promotes the process of carcinogenesis^28^.

Evidence available in many publications indicates a connection of the *TGFB1* gene with the pathogenesis of neoplastic diseases (among others, melanoma, lung or breast cancers), and therefore the authors of the study decided to assess the relative expression level of the gene in tissues of patients with colorectal cancer^29^. In the analysis, the relative levels of the *TGFB1* gene expression were compared with age, sex and family history of the patients as well as other clinical and pathological features. The most important aim of the study was to understand the mechanism of regulation of the *TGFB1* gene expression. To achieve this objective, the authors assessed the presence of methylation within the promoter region of this gene between − 235 and + 22 nucleotides from the start of transcription and then compared the data with demographic and clinico-pathological features.

## Materials and methods

The biological material for the research consisted of 64 tissue sections collected from patients with colorectal cancer during cancer removal surgery. All the analyzed tumor tissue fragments were confirmed by histopathological examination and obtained from the Department of Pathology, Medical University of Lodz, Poland. The material was frozen in liquid nitrogen immediately after collection and stored at − 80 °C until the analysis. Patients gave their informed consent to participate in the experiment and the research was conducted with the approvals of the Bioethics Committee of the Medical University of Lodz (RNN/214/00, RNN/8/08/KE, RNN/83/20/KE). All methods used in this study were performed in accordance with the relevant guidelines and regulations.

### RNA and DNA isolation

Total RNA and DNA were isolated from frozen tissue fragments of colorectal cancer using the column method according to the Total RNA Mini and Genomic Mini protocols (*A&A Biotechnology*, Poland). The concentration and purity of the RNA samples after isolation were measured spectrophotometrically using the NanoPhotometer™ (*IMPLEN*, Germany). RNA samples with a calculated A 260/280 absorbance value ranging from 1.8 to 2.0 were selected to perform the reverse transcription reaction. Isolated DNA from tissues showing adequate purity was stored for further analysis.

### RNA analysis—assessment of the level of gene expression

#### Reverse transcription reaction

At the next stage, the information contained in the isolated RNA was transcribed into complementary DNA. The reverse transcription reaction was performed using the *High Capacity cDNA Reverse Transcription Kit* (*Applied Biosystems*, USA). A final RNA concentration of 0.05 µg/µl was used in each sample prepared for the reaction. The composition of the reaction mixture and the temperature conditions for the reaction were provided in accordance with the manufacturer's recommendations.

### Qualitative analysis of *TGFB1* gene expression

To establish whether cDNA was obtained as a result of RT-PCR, the fragment of *GAPDH* gene was amplified by PCR. After confirming the correctness of the RT-PCR reaction, a qualitative evaluation of the *TGFB1* gene expression was performed using the PCR method. All PCR reactions were carried out on an MJ Mini™ Personal Thermal Cycler (*Bio-Rad*, USA). The sequence of the primers used in the PCR for *GAPDH* were 5′-TGGTATCGTGGAAGGACTCATGAC-3′ and 5′-ATGCCAGTGAGCTTCCCGTTCAGC-3′ and 5′-AACCCACAACGAAATCTATG-3′ and 5′-CTTTTAACTTGAGCCTCAGC-3′ for *TGFB1*. The composition of reaction mixtures and PCR reaction conditions for the analyzed genes are presented in Tables 1 and 2. For each PCR reaction, a negative control, containing the reaction mixture and nuclease-free water without the cDNA template, was run. The presence of the PCR products was confirmed by electrophoresis on a 2% agarose gel using a non-specific ethidium bromide dye, which allowed visualization of the PCR products under UV light. The product size for the *GAPDH* gene was 189 bp and the size was 146 bp for the *TGFB1* gene. Only samples which qualitatively showed presence of the tested gene were subject to further quantitative analysis.

**Table 1:** Composition of mixtures for PCR reactions.

Reagent	Volume [μL]
	*GAPDH*/*TGFB1*
Ready-to-use *2xPCR Super Master Mix* (*Biotool, Germany*) with buffer, dNTPs (conc. 0.5 mM), MgCl_2_ (conc. 4 mM), *Taq* DNA polymerase (conc. 100 U/ml)	5.0
Forward primer (conc. 10 μM)	0.7
Reverse primer (conc. 10 μM)	0.7
cDNA	1.0
Distilled water	12.6
Final volume	20.0

**Table 2:**
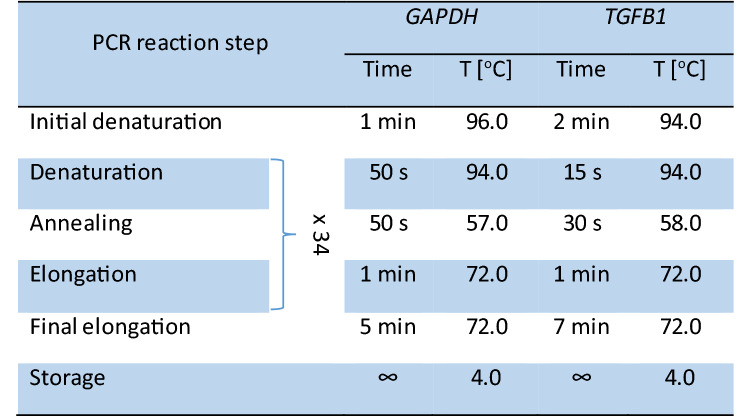
PCR reaction conditions for the reference (*GAPDH*) and the study (*TGFB1*) gene.

### Quantitative analysis of *TGFB1* gene expression—real-time PCR

The real-time PCR technique was applied to quantify the level of mRNA expression of the *TGFB1* gene. The amplification reaction was performed on a Mx3005P QPCR SYSTEM (*Stratagene*, USA) using the *SYBR*^*®*^* Green JumpStart Taq Ready Mix* reagent kit (*Sigma Aldrich*, Germany). For the analyzed samples, the *TGFB1* gene and the *GAPDH* reference gene were amplified parallelly, in separate tubes. Each reaction was performed in triplicate. The negative control, devoid of template cDNA, was amplified in each cycle of real-time PCR reactions. The reaction mixture for one sample was composed of 7.5 µl of the ready-made reagent mix containing buffer, MgCl_2_ (7 mM), dNTPs (0.4 mM) and *Taq* polymerase (0.05 U/μl); 0.7 µl of each primer for the *TGFB1* gene or *GAPDH* gene at a concentration of 10 µM and 1 µl of cDNA and nuclease-free water to a final volume of 16 µl. The same primers were used for both real-time PCR and the qualitative analysis. The time–temperature profile of the real-time PCR reaction included an initial denaturation step at 95 °C for 10 min, then 40 cycles, each of which consisted of three steps, i.e. a 30-s denaturation at 95 °C, 1-min annealing of primers to the template at 59 °C and 1-min extension of the template at 72 °C. After all the reaction cycles were completed, there was a final primer annealing step for 30 s at 55 °C and a final denaturation for 30 s at 95 °C.

**Figure 1:**
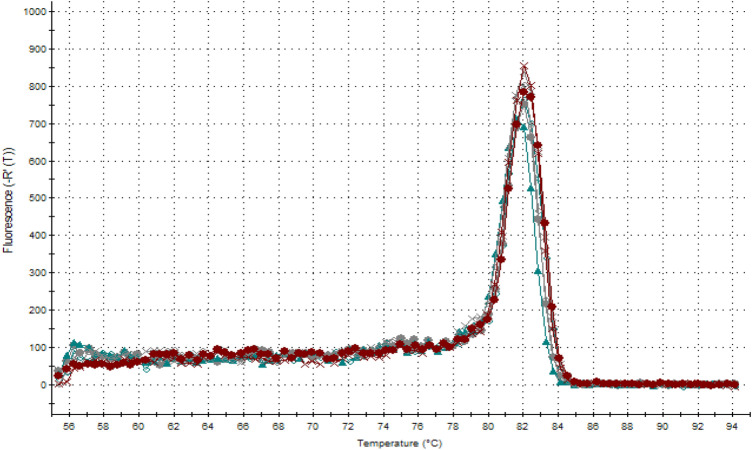
The melting curves of the real-time PCR amplification products of the *TGFB1* gene.

Due to the use of a nonspecific fluorescent dye, *SYBR Green*, which has the ability to intercalate between each double-stranded DNA molecule as a marker, it was necessary to carry out a reaction specificity control. To this end, after each real-time PCR reaction, the process of denaturation of the obtained amplification products was performed and the specificity of these products was assessed based on the obtained melting curves. The amplified products had a characteristic melting temperature (T_m_ for *GAPDH* gene = 88 °C, T_m_ for *TGFB1* gene = 81 °C) resulting from their nucleotide sequences. An example of a melting curve plot for the *TGFB1* gene was shown in Fig. 1.

Based on the obtained amplification diagrams, the C_t_ point was determined for each sample, which corresponded to the moment when the reaction entered the phase of the logarithmic increase in the amount of the product. The kinetic efficiency of real-time PCR was estimated based on the analysis of the standard curves of the *GAPDH* and *TGFB1* genes. For this purpose, from one of the tests following the PCR reaction, for which the concentration of the obtained product was also determined, a series of dilutions ranging from 10 to 10^7^ were prepared. Then, real-time PCR was performed for each of the dilutions and the C_t_ values were read. They were used to determine the reaction efficiency. It was calculated from the slopes of the standard curves according to the equation E = 10 [− 1/slope] − 1. PCR efficiency differed between the compared genes significantly (111% for *GAPDH* and 101% for *TGFB1*), therefore the Pfaffl's method was used to calculate the relative expression ratio, taking into account the level of reaction efficiency for each of the genes.

### DNA analysis—assessment of methylation of the CpG sequence of the *TGFB1* gene

#### Methylation-specific PCR

**Figure 2:**
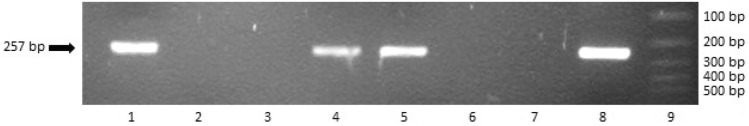
Image of gel electrophoresis of MS-PCR products. 1, 4, 5—methylated samples, 2, 3, 6—unmethylated sample, 7—negative control (unmethylated), 8—positive control (methylated), 9—molecular-weight size marker. Original gel is presented in Supplementary Fig. 1.

The assessment of the CpG island methylation of the promoter region of the *TGFB1* gene required a stage of DNA purification and preparation for further studies. For this purpose, the conversion of unmethylated cytosines to uracil was performed according to the *CiTi Converter DNA Methylation Kit* protocol (*A&A Biotechnology*, Poland). The MS-PCR reaction was subsequently performed using the modified *Taq-CiTi HotStart DNA polymerase*, which prevents the extension of the template containing the modified nucleotide. The reaction was carried out with the use of the *CiTi Converter MSP PCR Kit* (*A&A Biotechnology*, Poland). The composition of the reaction mixture for MS-PCR amplification for one sample was the following: a ready-to-use 2x*CiTi MSP PCR Mix* (*A&A Biotechnology*, Poland) in the amount of 25 µl, forward and reverse primers in the volume of 1.75 µl each, DNA after conversion in the amount of 2.5 µl and sterile water to a final volume of 50 µl. The MS-PCR reaction was carried out in a thermocycler in controlled temperature–time conditions, with an initial denaturation of the template and activation of *CiTi HotStart DNA polymerase* for 5 min at 95 °C, followed by 35 cycles consisting of three repeated steps, i.e., denaturation for 15 s at 95 °C, primer annealing for 30 s at 59 °C and extending the template for 30 s at 72 °C. In the final stage sample was incubated in 10 °C for 10 min. The evaluation of the CpG sequence of the *TGFB1* promoter region gene was performed based on electrophoretic separation of the MS-PCR reaction products (Fig. 2). The product size was 257 bp.

### Bioinformatic analysis of promoter region methylation

TCGA data available in the UALCAN database (http://ualcan.path.uab.edu/index.html) was used to compare the promoter methylation level in the colon adenocarcinoma and healthy colon tissue. The beta value is the ratio of the methylated probe intensity and the sum of methylated and unmethylated probe intensity. Beta value ranges from 0 (unmethylated) to 1 (fully methylated). The boxplot represents beta values of CpG probes located up to 1500 bp upstream of gene's start site.

## Results

The *STATISTICA* 13.1 software (*StatSoft Inc.*, USA) was used to statistically evaluate the obtained results.

The first assessment focused on a comparison between the age of the patients in the study group at the time of developing colorectal cancer and the relative mRNA level of the *TGFB1* gene. The median age in the studied group of patients was 63 years (57 years for women and 66 years for men), the youngest patient was 34 years old and the oldest subject was 82 years old. The analysis of dependence did not show any correlation between the compared features (*p* = *0.1380*).

The second step of the statistical evaluation was to compare the relative expression level of the studied gene with respect to sex. Men are statistically more likely to develop colorectal cancer than women. For this reason, it becomes justified to search for molecular bases that may contribute to the emergence of differences in the incidence of this type of cancer. The study compared the relative level of *TGFB1* gene expression in the group of 30 women and 20 men, and no statistically significant differences were observed (*p* = *0.9763*).

Some cases of colorectal cancer are hereditary, therefore, in the next stage of the statistical analysis, an association between the relative level of *TGFB1* gene expression and presence of cancer in a patient's immediate family was determined. The percentage of patients confirming presence of colorectal cancer in the family was 33%, while a majority of the patients included in the study came from families in which cases of colorectal cancer had not been previously diagnosed. Moreover, no statistically significant correlation was found between family history and the relative level of *TGFB1* gene expression (*p* = *0.8149*).

Due to different therapeutic procedures and for prognostic reasons, the location of neoplastic lesions is important. Therefore, another assessed feature was the correlation of the relative expression level of the studied gene with the location of colorectal cancer. Forty nine patients included in this analysis were divided into two groups, i.e., those with cancer of the rectum (20 patients) and those with cancer located in the upper sections of the large intestine (29 patients). However, no statistically significant correlation was found between the relative level of *TGFB1* gene expression and cancer location (*p* = *0.1246*).

Another assessed feature was the histopathological subtype of colorectal cancer. Based on histopathological examination, three different types of adenocarcinomas were distinguished in the studied population, i.e., adenocarcinoma tubulare (32 patients), adenocarcinoma mucinosum (9 patients) and adenocarcinoma tubulare/mucinosum (6 patients). In each subgroup, the expression level of the analyzed gene was compared. The comparative analysis showed no differences between the relative level of *TGFB1* gene expression and the histopathological type of cancer (*p* = *0.9561*).

Based on the microscopic evaluation, each tumor tissue collected from the study group patients was classified into one of three categories, G1, G2 or G3, depending on the degree of histological differentiation of cells. Most of the observed cases were tumors with moderately (G2) and poorly differentiated cells (G3) (92%). Due to the small number of well-differentiated adenocarcinomas (G1), groups G1 and G2 were combined for statistical purposes. The analysis showed that the relative expression level of the *TGFB1* gene did not differ between the groups (*p* = *0.1102*). Despite the lack of a statistically significant association between the transcript level of the *TGFB1* gene and the degree of histological malignancy, a tendency was noticed to achieve higher relative expression levels of this gene by G1 and G2 adenocarcinomas as compared to G3.

**Figure 3:**
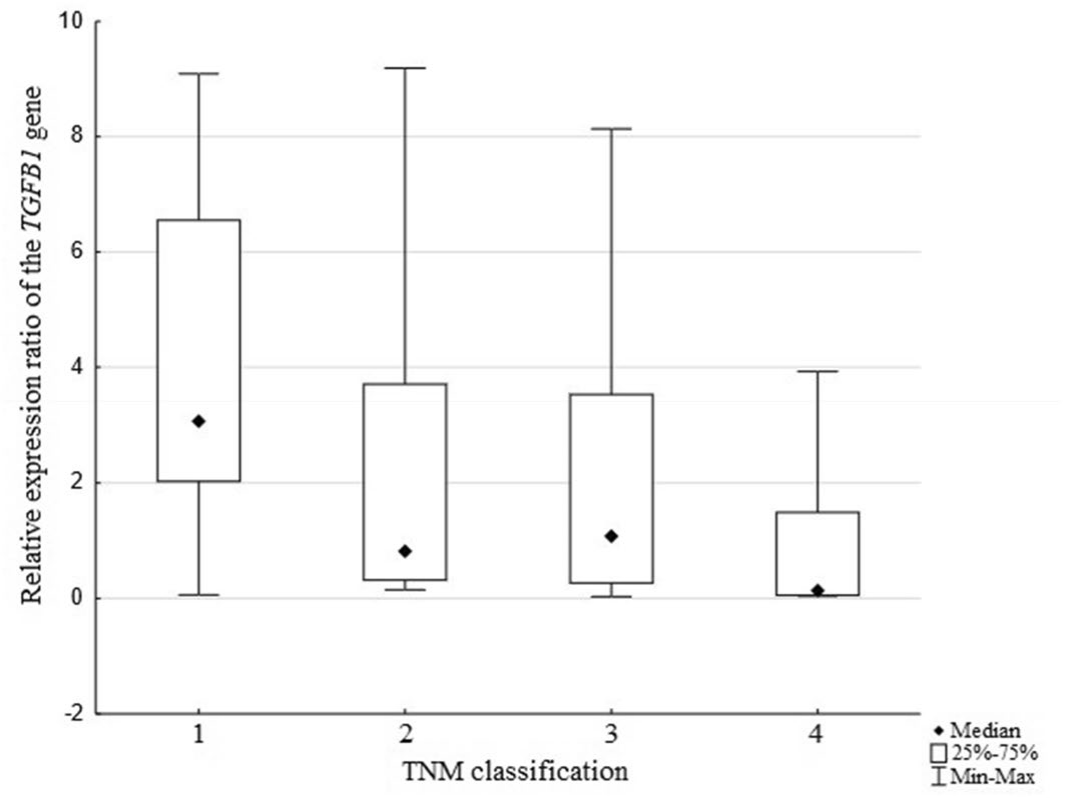
Relative expression levels of the *TGFB1* gene and clinical stage according to the TNM classification.

In order to conduct another comparative analysis, the study group was divided into four subgroups based on the clinical stage of cancer according to the TNM classification (I—16 cases, II—12 cases, III—13 cases, IV—9 cases). The statistical analysis showed that the relative level of *TGFB1* gene expression differed depending on the stage of clinical advancement, and that association was statistically significant (*p* = *0.0113*, Fig. 3). It may indicate a potential influence of this gene on the course of carcinogenesis in the large intestine. Moreover, a statistically significant difference in the relative levels of *TGFB1* gene expression was demonstrated between TNM grades I and IV (*p* = *0.0090*). Higher relative transcript levels of the *TGFB1* gene were observed in stage I patients as compared to stage IV patients. The obtained results indicate that lowering the level of *TGFB1* gene expression may be one of the factors contributing to the progression of colorectal cancer.

In the next stage, an association between the individual TNM classification parameters and the expression of the *TGFB1* gene was assessed. The first analyzed parameter was the T feature, indicating the size of the primary lesion and tumor penetration of the intestinal wall. A correlation was observed between the relative mRNA levels of the *TGFB1* gene and the T feature of the TNM classification, and the association was statistically significant (*p* = *0.0206*). The highest relative expression levels were found in the T1 and T2 stages, which may indicate the inhibitory effect of this gene expression on the size and depth of tumor infiltration. Another assessed parameter of TNM classification was the feature N, which indicates presence of neoplastic cells in the regional lymph nodes. The applied test showed no statistically significant differences in the level of *TGFB1* gene expression in different N stages of TNM classification (*p* = *0.4884*). The last element of TNM classification was the feature M indicating presence or absence of distant metastases. It was found that the relative transcript levels of the *TGFB1* gene differed in the subjects depending on presence of distant metastases (*p* = *0.0120*). The patients without diagnosed metastases were characterized by higher relative expression levels of the analyzed gene, which may indicate a protective role of *TGFB1* in the process of metastasis formation.

**Figure 4:**
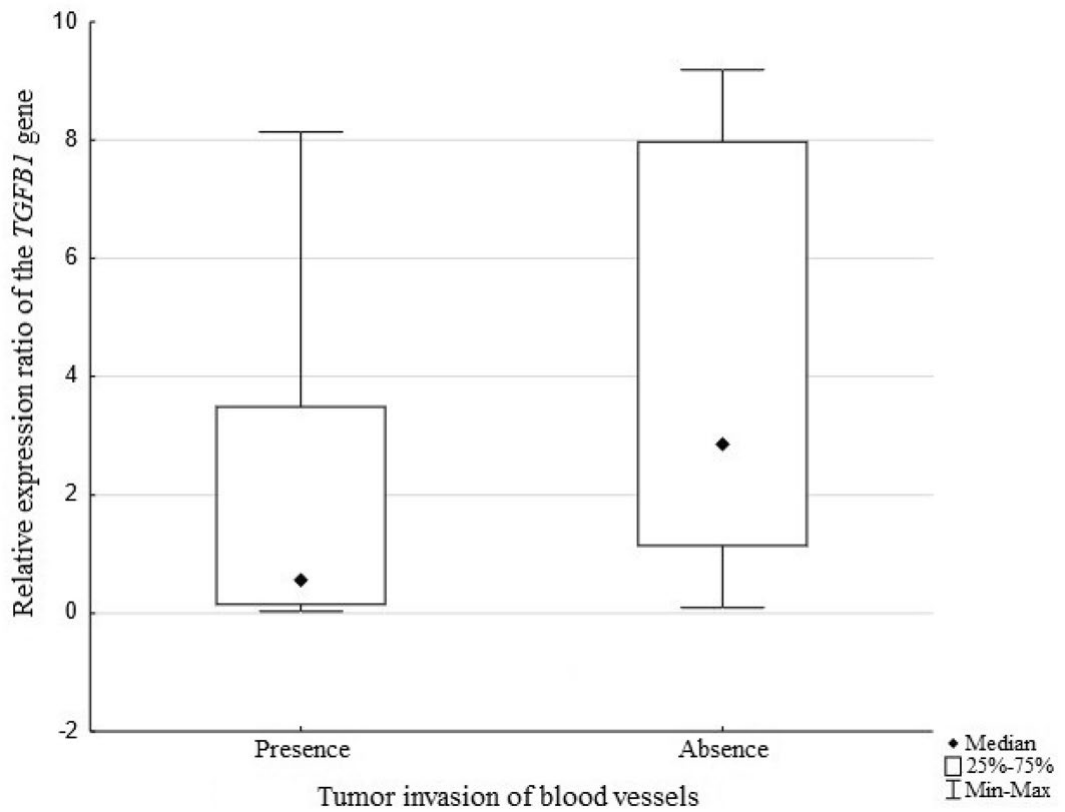
Relative expression levels of the *TGFB1* gene and tumor invasion of blood vessels.

Histopathologically confirmed presence of neoplastic cells in blood vessels is an unfavorable prognostic factor in the course of colorectal cancer. The statistical analysis showed an association between the relative levels of *TGFB1* gene expression and tumor invasion of blood vessels (*p* = *0.0125*) which is presented in Fig. 4. Higher relative transcript levels of the *TGFB1* gene were found in cancer cases where blood vessels were not affected by neoplastic cells, which may indicate that the studied gene plays a role in preventing the formation of secondary neoplastic lesions in distant organs.

**Figure 5:**
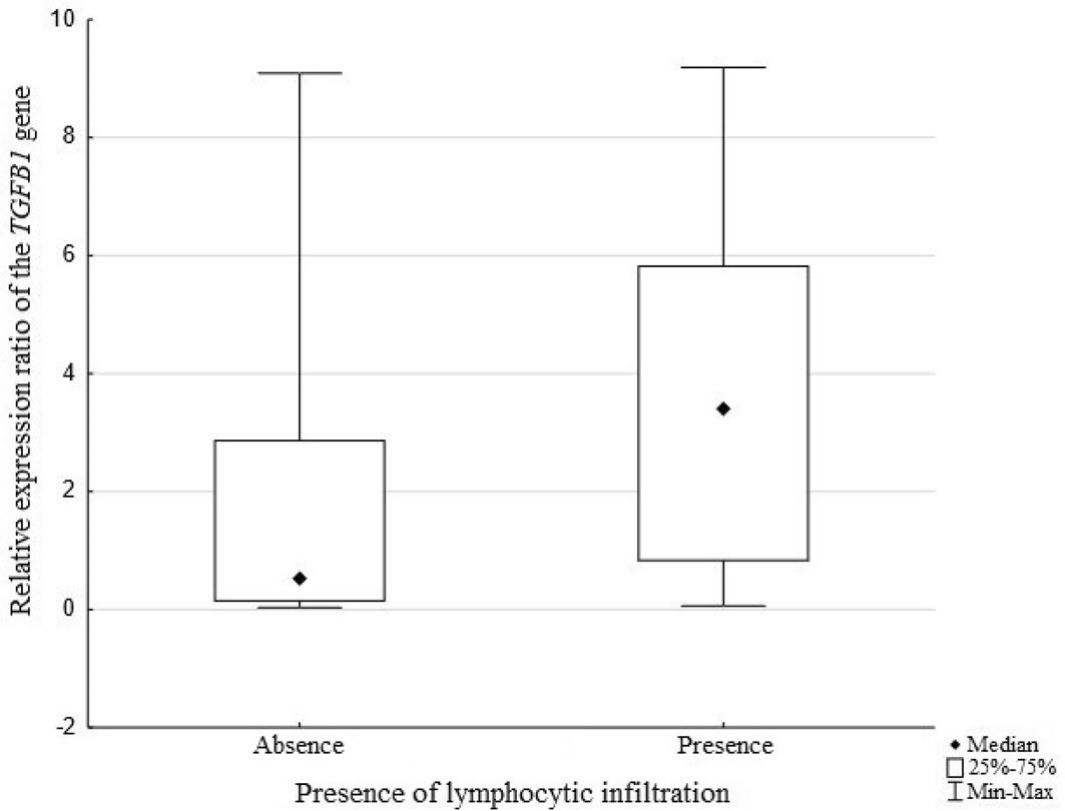
Relative expression levels of the *TGFB1* gene and presence of lymphocytic infiltration in the tumor tissue.

The presence of lymphocytic infiltration in the tumor mass is a positive prognostic indicator of advanced colorectal cancer. For this reason, another analysis was made to check an association between the expression of the studied gene and presence of lymphocytic infiltration in the neoplastic tissue of the colon. The analysis shows that there is a statistically significant association between the compared parameters (*p* = *0.0087*, Fig. 5).

It has been shown that higher relative levels of *TGFB1* gene expression are observed more frequently in patients with colon cancer accompanied by lymphocytic infiltration. It may indicate an important role of the *TGFB1* gene in regulating the immune response against cancer.

The survival analysis of the patients did not reveal any statistically significant differences in survival times depending on the relative expression level of the studied gene (*p* = *0.5257*).

All results concerning the evaluation of the *TGFB1* gene expression in colorectal cancer tissues were collected and presented in Table 3.

**Table 3:** Characteristics of the study group and *p*-values for the analysis of the *TGFB1* gene expression in relation to clinical-pathological features.

Characteristics	N	*p*
Age	50	0.1380
**Sex**		
Female	30	0.9763
Male	20	
**Family history**		
Positive	6	0.8149
Negative	12	
**Tumor location**		
Rectum	20	0.1246
Cecum or colon	29	
**Histopathological subtype**		
Adenocarcinoma tubulare	32	0.9561
Adenocarcinoma mucinosum	9	
Adenocarcinoma tubulare/mucinosum	6	
**Histological differentiation**		
G1 or G2	33 (4 + 29)	0.1102
G3	17	
**TNM classification**		
Stage I	16	0.0113
Stage II	12	
Stage III	13	
Stage IV	9	
**Size of tumor (T feature)**		
T1 or T2	17 (6 + 11)	0.0206
T3	27	
T4	6	
**Regional lymph node involvement (N feature)**		
N0	30	0.4884
N1	8	
N2	10	
**Presence of distant metastases (M feature)**		
M0	41	0.0120
M1	7	
**Blood vessels involvement**		
Presence	35	0.0125
Absence	15	
**Lymphocytic infiltration**		
Presence	20	0.0087
Absence	30	
Survival	40	0.5257

**Figure 6:**
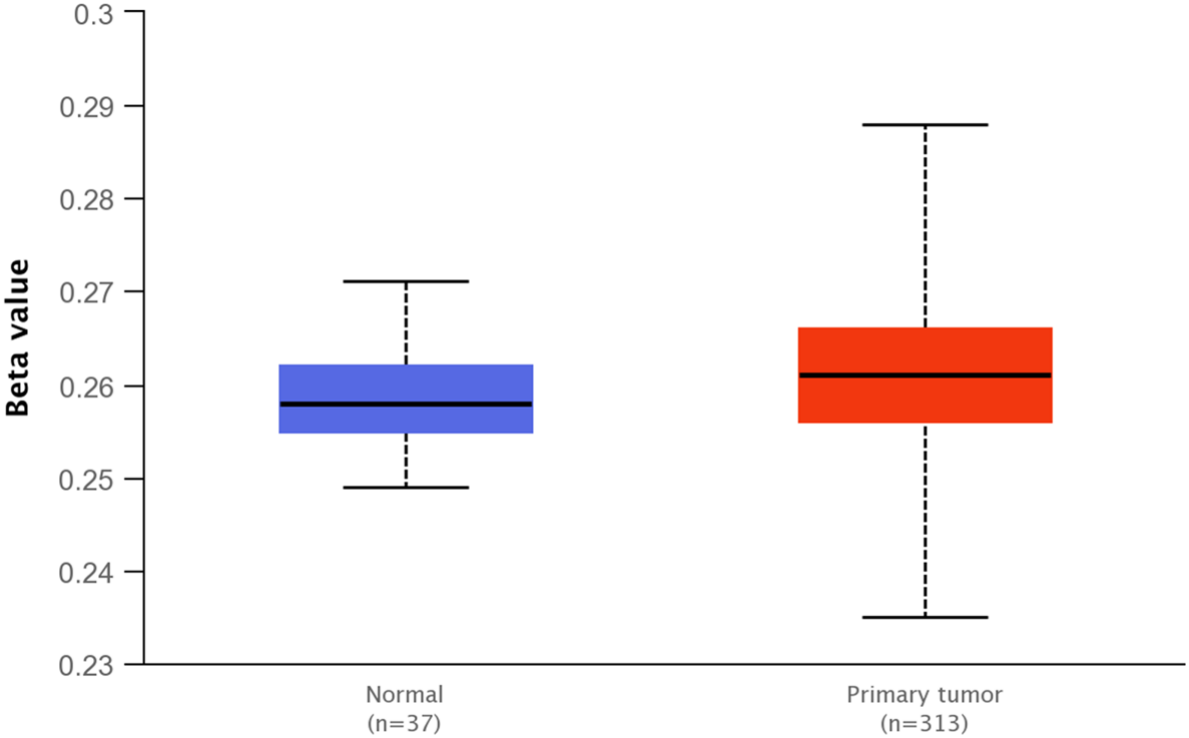
The promoter methylation level of the *TGFB1* gene in colon adenocarcinoma tissue in comparison with normal colon tissue by the UALCAN database.

The observed differences in the expression levels of the *TGFB1* gene in the study group meant that the next goal of the study was to understand the mechanism of regulation of this gene expression. Since DNA methylation is one of them, the frequency of occurrence of methyl groups within the promoter region of the *TGFB1* gene in normal and neoplastic tissues was compared using the UALCAN bioinformatics tool (http://ualcan.path.uab.edu/index.html). The analysis showed that the promoter methylation level was substantially higher in colorectal cancer in comparison with normal tissues (*p* < *0.001*, Fig. 6).

Therefore, the next step of the analysis was to check the methylation status in the selected area of the promoter region and refer it to the demographic and pathological parameters of the patients as well as the expression level of the studied gene. In the investigated group, methylation of the promoter region of the *TGFB1* gene was confirmed in 26 cases (40.6%), whereas 38 of them (59.4%) were devoid of 5-methylcytosines in the observed area. The mean age of the patients with confirmed methylation within CpG islets preceding the *TGFB1* gene was 59 years and it was lower than the mean age of the patients without methylation (62 years), however, that difference was not statistically significant (*p* = *0.2894*). The study population included 32 women and the same number of men. In both groups, an advantage was observed in the patients without methylation of the promoter region of the *TGFB1* gene. The statistical analysis did not confirm any statistically significant association between the compared features (*p* = *0.6107*). Out of the 63 patients, 40 had cancer located in the caecum or colon and 23 had cancer of the last section of the large intestine. The comparison did not show any statistically significant association between the frequency of methylation in the promoter region of the *TGFB1* gene and the location of colorectal cancer (*p* = *0.1860*). Within the study group, most of the 61 patients had tubular adenocarcinomas (N = 44), while mucinous and tubulo-mucinous adenocarcinomas were sparse (N = 8 and N = 9, respectively). There was no significant correlation between the frequency of methylation within the promoter region of the *TGFB1* gene and the histopathological subtype of colorectal cancer (*p* = *0.1606*). Comparing the frequency of methylation with the degree of histological malignancy of cancer cells, a higher frequency of methylated cytosines in the promoter region of the *TGFB1* gene was observed in poorly differentiated neoplasms as opposed to cancers with a good and moderate degree of cellular differentiation. The performed statistical analysis did not confirm the association between G1 and G2 groups and G3 group (*p* = *0.0806*). The evaluation, based on a comparison of the frequency of methylation of the *TGFB1* gene promoter region with the clinical advancement according to the TNM classification, showed a more frequent methylation of the region regulating *TGFB1* gene expression in the most advanced stage III and IV cancers (56% and 53%, respectively) as compared to cancers in earlier stages I^o^ and II^o^ (33% and 19%, respectively). However, the association turned out to be statistically insignificant (*χ*^2^ test, *p* = *0.1026*). Another analyzed feature was the invasion of blood vessels by the neoplasm. Despite the noticeable tendency to a higher frequency of methylation of the area under study in neoplasms with vascular invasion, the performed statistical analysis did not confirm any association between the compared features (*p* = *0.0505*). However, it may be assumed that methylation of the promoter region silencing *TGFB1* expression promotes the invasion of blood vessels by the tumor. Comparison of the frequency of cytosine methylation within the promoter region of the *TGFB1* gene with the presence of lymphocytic infiltration in the neoplastic tissue also showed no statistically significant correlation (*p* = *0.3624*). When comparing the survival of the patients depending on the methylation status of the CpG islets of the analyzed area, the survival time of the patients was not dependent on the presence of methylation within the promoter region of the *TGFB1* gene (*p* = *0.8000*). The final step in the statistical evaluation was to compare the methylation frequency of the promoter region of the *TGFB1* gene with the relative expression level of the gene. The performed analysis showed no differences in the levels of relative expression of the *TGFB1* gene and methylation (*p* = *0.2825*).

## Discussion

The transforming growth factor *β* signaling pathway is involved in many physiological cellular processes. Disturbances in genes expression within the TGF-*β* pathway are one of the factors involved in development of many cancers where they can act as both a suppressor and a promoter of carcinogenesis^29^.

The results obtained in the study are consistent with the phenomenon described as the TGF-*β* paradox. Numerous reports indicate that in the initial stages of tumorigenesis the TGF-*β* pathway acts as a tumor suppressor, however, as the disease progresses, it begins to play an oncogenic role^28,30^. It may be connected with the ability of the TGF-*β* to arrest the cell cycle in the G1 phase by inhibiting the activity of cyclin-dependent kinases such as p15, p21 and p57. Additionally, the activity of kinases is attenuated by the action of the TGF-*β* which reduces the synthesis of c-MYC^9,12^. The suppressor function may be less effective with an increase in the number of mutations in the genes encoding the proteins of the TGF-*β* pathway as well as in the target genes. As the number of mutations increases, the ability to remodel the extracellular matrix, synthesize chemokines, angiogenesis or avoid immune system control mechanisms is also acquired. At the same time, the oncogenic activity of the TGF-*β* cytokine, e.g., signalling of the epithelial-mesenchymal transition, remains preserved^30^.

In the early stages of carcinogenesis, the high relative expression level of the *TGFB1* gene may be an element of the suppressor response preventing the fixation of genetic changes in the cell. On the other hand, decreased level of the *TGFB1* gene expression during the course of the disease observed in this study, may be an important result in the pathogenesis of colorectal cancer allowing for its further development. When studying the colon cancer cell line, Zhao et al. observed that TGF-*β*1 decreased the expression of the gene encoding VEGF and blocked the growth of enterocytes. It was also confirmed that TGF-*β*1 increased the apoptosis by inhibiting the COX-2 and PGE2 production^24^. This may suggest that the reduction of the *TGFB1* expression observed in the most advanced neoplasms is associated with loss of control over this process.

The suppressive effect of the TGF-*β* on normal cells has been documented in many studies, however, in neoplastic cells its role in promoting carcinogenesis is becoming apparent. The high relative expression levels of the *TGFB1* gene observed in the study in the least clinically advanced colorectal cancers may prove these findings. As reported by Suzuki et al., it may mean that inhibition of the TGF-*β* signaling is one of the most important factors for neoplastic cell proliferation^31^. The experiment with the breast cancer cell line shows that the TGF-*β* reduces the expression of the *ATM*, *MSH2* and *BRCA1* genes involved in the response to DNA damage. As a consequence, lowering the expression levels of these genes enables cells to escape from control mechanisms and allows their unlimited proliferation^9,32^. Moreover, studies conducted by Qin et al. on the prostate cancer cell line show that the TGF-*β* stimulates the process of carcinogenesis already in the initial stages of tumorigenesis^33^. For this reason, the obtained high expression of the *TGFB1* gene in patients with low-advanced colorectal cancer may be a negative prognostic indicator of the disease course. Furthermore, selective inhibition of the TGF-*β* transmission in its early stages could prove to be a new therapeutic strategy.

In a study conducted in women diagnosed with breast cancer, an increased amount of the *TGFB1* transcript promoted development of cancer stem cells and additionally improved their resistance to chemotherapy^25^. An increase in the cells mobility and a higher likelihood of metastasis were noted after radiotherapy in another group of breast cancer patients. It may be explained by sensitivity of cancer cells to radiation, as a result of which the TGF-*β*1 is released from LLC complexes. However, it is also very important that blocking the function of this protein eliminated such effects^34^. There is evidence proving that a dysfunction or loss of TGF-*β* signaling promotes tumor progression and that enhancement of its transmission has a positive effect on prognosis and prevents progression of colorectal cancer in its early stages^35,36^. The discrepancy in the research results may be explained by the presence of cytokines in the neoplastic microenvironment which take the form of large protein conglomerates with an activity that is difficult to predict from the level of the gene transcript. Thus, the presence of inactive LLC complexes appears to be one of the ways modulating the TGF-*β* pathway transmission. An unquestionable limitation of the presented studies is a lack of information on the TGF-*β*1 protein activity. Moreover, when interpreting the results, it should be noted that increased *TGFB1* expression did not have to be equivalent to an increase in cytokine-initiated signaling.

An interesting observation in our study was proving the impact of the *TGFB1* gene expression on the presence of liver metastases. The obtained results are consistent with other reports on the involvement of the TGF-*β* pathway in the formation of metastases in neoplasms of the lung, thyroid, prostate, bladder, esophagus and colon^33,35,37–40^. Among the molecular bases influencing progression and the ability to form metastases, the action of TGF-*β* is believed to stimulate formation of new blood vessels by increasing the expression of vascular endothelial growth factor (VEGF) and connective tissue growth factor (CTGF)^31,41,42^.

There are many reports that the TGF-*β*1 cytokine is responsible for acquiring the features typical for mesenchymal cells which they acquire in the process of epithelial-mesenchymal transition. It is related to the increased expression of such transcription factors as SNAIL1/2 (human snail homolog 1/2), HMGA1 (high mobility group AT-hook 1), ZEB1/2 (zinc finger E-box binding homeobox 1/2) and TWIST1 (twist basic helix-loop-helix transcription factor 1)^10,28,43^. As a result, cells lose the function of E-cadherin and *γ*-catenin adhesion proteins in favor of increased expression of mesenchymal markers, i.e., vimentin and N-cadherin^40,44^. It promotes the loss of cells polarity and breaking the connections between them. Consequently, they acquire features typical of mesenchymal cells with greater mobility and ability to migrate^26,27^.

What is more, equally important observation in the study was related to the high relative expression levels of the *TGFB1* gene expression in neoplasms without blood vessel involvement. It is believed that high levels of the gene transcript can initiate vascular invasion during tumor development which is an unfavorable prognostic factor. The obtained results may confirm the contribution of the *TGFB1* in increasing the migratory capacity of cells and the formation of secondary cancer foci in distant organs. These actions are explained by the ability of the TGF-*β*1 to regulate the functions of integrins, e.g., *α*V, *β*1 and *β*3 and promoting the EMT process^26^. These reports indicate that the *TGFB1* affects intensification of proliferative processes and acquisition of the ability to form metastases. In the case of the studied patients, these processes could be initiated at the early stages of tumor development and precede the silencing of this gene transcription^45^.

Another interesting finding was also higher relative expression levels of the studied gene in the patients with lymphocytic infiltration in the tumor tissue. There are studies showing that overexpression of the *TGFB1* gene in neoplastic diseases blocks the anti-tumor immune response by suppressing lymphocytes T and B, NK cells and macrophages^28^. This is confirmed by a study conducted in patients with advanced colorectal cancer in whom the *TGFB1* overexpression was associated with a reduced number of T cells and a blockage of the acquisition of the Th1 phenotype by them^46^.

Other sources report that high expression of this cytokine inhibits the Th lymphocyte response and induces Treg lymphocytes expressing the Foxp3 transcription factor which suppress the immune response and promote tumorigenesis^47,48^. Increased infiltration of Treg lymphocytes within the tumor microenvironment may thus create a favorable niche for cancer development^49,50^. This would also explain the higher relative expression levels of the *TGFB1* in tissues with lymphocytic infiltration, which has been observed in this study. Our results do not contradict the current reports on the immunosuppressive role of the TGF-*β*1 since a large part of the lymphocytes present in the neoplastic tissue may be the Treg cells fraction^49^. However, to verify the hypothesis about the increased share of Treg cells in relation to Th cells, additional immunohistochemical tests of tissues are required to determine the phenotype of lymphocytes.

The mechanisms regulating gene expression include epigenetic factors, such as modifications of histone proteins or methylation of cytosines within the promoter and regulatory regions of genes. To investigate the regulation of the expression of the studied gene, a qualitative evaluation of the CpG island methylation of the *TGFB1* promoter region between − 235 and + 22 nucleotides from the transcription start was performed. The presence of methyl groups within the analyzed region was confirmed in 40.6% of the patients, which was similar to the values obtained by another team that found methylation in 44.0% of lung cancer cases. However, these results were significantly different from those observed in prostate cancer patients with a methylation rate of 82.0%. Moreover, Shah et al. noted that a higher frequency of the CpG islands methylation is characteristic for invasive prostate cancers and metastatic lung tumors^51^.

It should be emphasized that methylation did not have to occur only in the analyzed area, as it could have taken place simultaneously in other regulatory sites. Therefore, the next stages of the research should exclude the possibility of adding methyl groups within remaining regions influencing the expression of the analyzed gene (e.g. at positions + 91, + 115, + 145 and + 151 from the transcription start site)^52^. It is worth considering the role of other epigenetic factors that may affect the expression of the *TGFB1* gene, including post-translational modifications of histone proteins and the participation of non-coding RNA molecules, such as siRNA and microRNA.

The available scientific data indicate the association of the TGF-*β* cytokine with the development of many diseases, including cancers. The results presented in the study prove a potential involvement of the *TGFB1* gene in the development of colorectal cancer and its progression, however, they should be confirmed in a larger group of patients. Since this experiment did not include an analysis of the immunophenotype of lymphocytes in tumor tissue, assessment of methylation in the remaining regulatory regions of the *TGFB1* gene or an impact of other epigenetic factors affecting the level of expression of this gene, such analyzes should be included in subsequent studies. Additional experiments will ensure a better understanding of the mechanisms regulating expression of this gene in the future. These outcomes can be further used to identify epigenetic markers for colorectal cancer or become the target of personalized therapy.

## Conclusions

The decreased expression of the *TGFB1* gene found in colorectal cancer with high clinical advancement stages indicates that the loss of the physiological function of the gene and the protein it encodes is conducive to disease progression. The protective role of the *TGFB1* gene is confirmed by higher expression levels obtained in patients whose blood vessels are not affected by tumor cells and in presence of lymphocytic infiltration in cancer tissue, which are positive prognostic factors for colorectal cancer. The results presented in the study did not confirm an association between methylation of the assessed area and the clinical or pathological features of the patients. The most important conclusion was that the methylation of the studied area had no effect on the expression level of the *TGFB1* gene.

## Supplementary Information

Supplementary Figure 1.
