# Supplementary material for: Assessment of the TGFB1 gene expression and methylation status of the promoter region in patients with colorectal cancer
Source: Sci Rep. 2022 Jul 7;12:11488. doi: 10.1038/s41598-022-15599-4 (PMC9263105; doi:10.1038/s41598-022-15599-4)
Supplement: Supplementary file 1 — Supplementary Figure 1. [file 41598_2022_15599_MOESM1_ESM.docx]

**Supplementary figure**


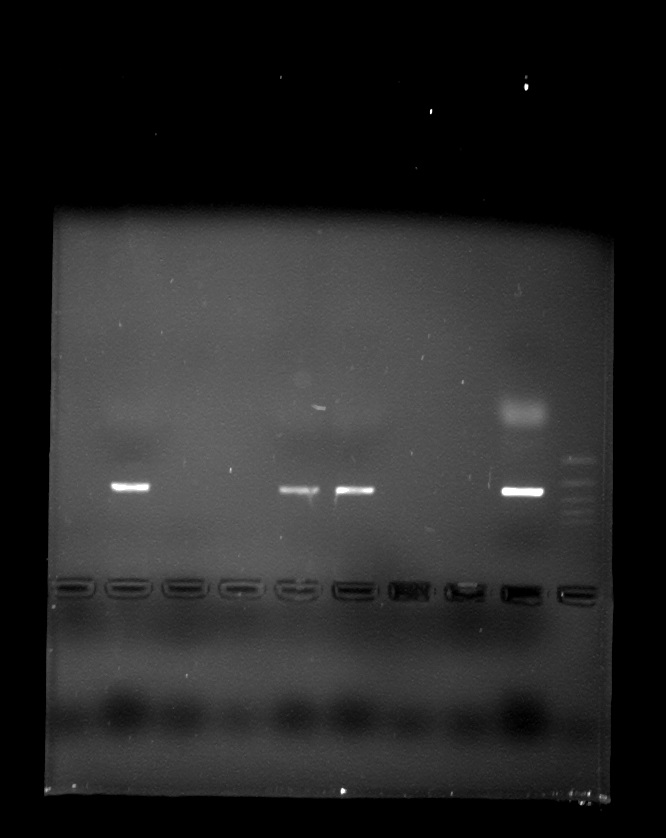


Supplementary Figure 1. Image of gel electrophoresis of MS-PCR products obtained under UV light using the *MiniBis Pro* analyzer (*DNR Bio-Imaging Systems*, Israel) and the *Gel Capture* program.
